# Pediatric Motor Vehicle-Pedestrian Accident: a Simulation Scenario for Emergency Medicine Trainees

**DOI:** 10.7759/cureus.1052

**Published:** 2017-02-23

**Authors:** Sarah Mathieson, Kerry-Lynn Williams, Adam Dubrowski

**Affiliations:** 1 Emergency Medicine, Memorial University of Newfoundland; 2 Faculty of Medicine, Memorial University of Newfoundland; 3 Emergency Medicine, Pediatrics, Memorial University of Newfoundland

**Keywords:** emergency medicine, pediatric emergency medicine, simulation-based medical education, pediatric trauma

## Abstract

Simulation-based medical education is an evolving field that allows trainees to practice skills in a safe environment with no risk to patients. Recently, technology-enhanced simulation for emergency medicine learners has been shown to have favorable effects on learner knowledge and patient outcomes. In this report, a human patient simulator is used to familiarize emergency medicine trainees with the presentation and management of a pediatric motor vehicle-pedestrian accident is described.

## Introduction

Trauma is the leading cause of death and disability in children greater than one year of age [1]. Despite this fact, it is still a relatively uncommon event and many trainees do not get adequate exposure to it without the use of simulation [2]. Much of the available literature indicates that the repeated use of high-fidelity simulation can improve the acquisition of knowledge, skills, and attitudes, as well as their translation to the clinical setting in relation to neonatal and pediatric resuscitation, Cardiopulmonary resuscitation (CPR) and procedural skills, including airway management [3-4]. “Procedural skills that used to be taught with the “see one, do one, teach one” approach are now being taught with a safer approach: “see one, simulate a lot, do one, teach one” [4]. 

Pediatric trauma can present the emergency room physician with challenges that are unique compared to adult trauma. Firstly, trauma in the pediatric setting is emotionally charged. Secondly, due to differences in anatomy and physiology in children compared with adults, injury management also differs in some regards. For a physician not working primarily in a pediatric trauma center, these differences may add an element of discomfort when dealing with the injured child. Although many injuries can be managed initially in a general emergency department, more seriously injured children require transportation to a designated pediatric trauma center.

The use of simulation can help to familiarize residents, as well as practicing physicians, with the various elements of pediatric trauma, thereby improving performance in real-life situations. Simulation is a useful tool to improve medical skills and knowledge. It also addresses the less tangible aspects of running a trauma; for example teamwork [5], dealing with death or significant morbidity and emotional reaction of the healthcare team. This is especially important in the pediatric setting. 

## Technical report

This in-situ simulation training session was conducted in the emergency department at the Janeway Children’s Health and Rehabilitation Centre using a high-fidelity mannequin simulator. This particular simulation used Laerdal SimJunior™ (232-05050).

**Figure 1:**
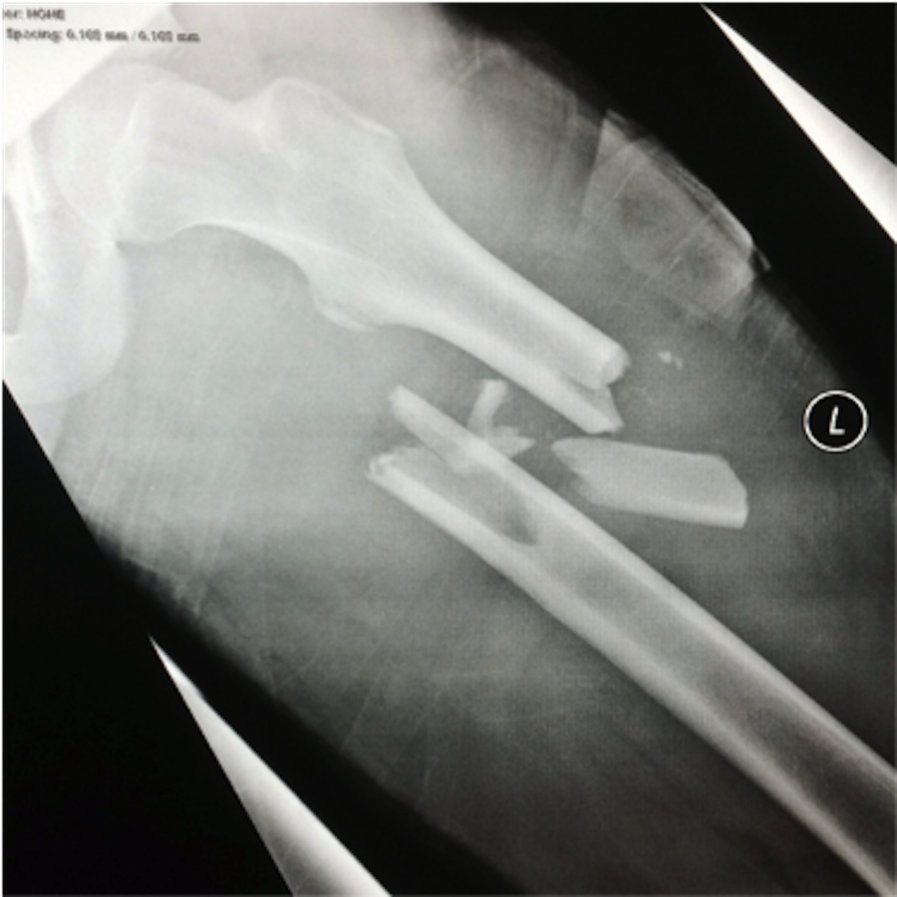
Radiograph of left femur demonstrating a comminuted mid-shaft fracture (Source: K Chan)

**Figure 2:**
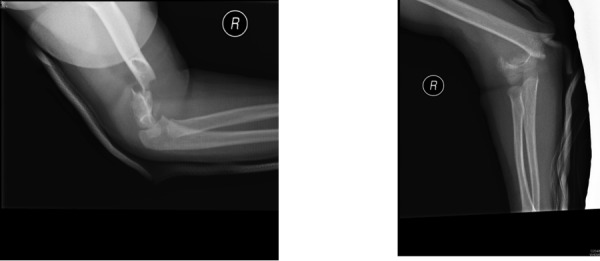
Radiographs of the right elbow demonstrating a supracondylar fracture (Source: K Chan)

Prior to the session, a comprehensive stepwise scenario template was developed (Table 1). This process required compiling all of the relevant clinical data and supporting documents, such as x-rays (Figures 1, 2) and laboratory data, that were to be used during the scenario execution. This was submitted to the simulation laboratory technical staff who then programmed the mannequin and supplied any necessary materials for the simulation.

Table 1

**Table 1:** A stepwise, detailed scenario template was submitted to the simulation lab’s technical staff, who then programmed the mannequin and supplied the necessary materials for the case

Pre-Scenario		
You are working in a rural emergency department. An eight-year-old male is brought in by ambulance after being struck by a pick-up truck as he ran across the road. He has fractures to his left femur and right elbow as well as various abrasions.The nearest trauma center is 90 minutes by road.		
History		
Allergies	Peanuts	
Medications	Flovent™, prn Ventolin™	
Past Medical Hx	Asthma (mild)	
Initial Vitals	T 36.5 (axillary) // HR 120 (sinus) // BP 100/65 // RR24 // spO2 100% RA // glucose 6 (as per EMS) The patient is alert, crying, anxious and in pain, doesn't want to talk to anyone but his father; collared and boarded by EMS	
HEENT	Laceration to R cheek	
CNS	Normal apart from anxious appearance	
Chest	Heart sounds normal, breath sounds normal bilaterally, trachea midline no obvious contusions or deformity	
Abdomen	Soft; non-tender	
Extremities	Deformed L femur, open wound; swollen and deformed R elbow; abrasions to R hip and arm; contusions to L hip and thigh	
Objective 1: Develop an approach to pediatric trauma		
Stage 1: Initial Assessment / Stabilization		
Stage	Vitals and Results of Investigations	Expected Action
Connect to cardiac monitor, oxygen saturation monitor, get new set of vitals, place 2 large bore IVs, assess ABCDE	A – airway patent and protected B – RR 24, no respiratory distress, clear air bilaterally, trachea midline C – BP 100/65, HR 120, heart sounds normal, peripheral pulses strong x4 limbs, abdomen soft and non-tender, obvious deformity and overlying open wound to L femur D – alert and oriented x3, power/tone/sensation/reflexes normal x4 limbs, pupils equal and reactive E – undress and logroll child while maintaining c-spine precautions to remove backboard and examine spine	
Get new set of vitals	Same as EMS	Start two large bore IVs
Without fluid resuscitation	HR increases to 130	
With father's presence	RR to 16, child calmer	Pain control should be given, second bolus given. After second bolus, should consider O^-^ blood if not stable
If adequate fluid	Vitals stable	Continue to stage two
If inadequate fluid	HR 135 // BP 85/50	Continue to stage two
Order investigations and labs		CBC, electrolytes, BUN, creatinine, liver enzymes, amylase, coagulants, T&S, crossmatch), x-rays (CXR, pelvis, c-spine, L femur and R elbow)
Stage 2: Mom arrives, visibly upset and agitated		
Mom addressed by lead and promptly assigned a chaperone (nurse* or *other)	Vitals stable	Continue on to stage three
No one promptly addresses Mom's anxiety	Child's anxiety increases. HR increases by 5/min, RR 22, the child starts crying again	Address Mom, and then continue on to stage three
Objective 2: Recognize common pediatric injuries and their appropriate management		
Stage 3: Secondary Survey		
Secondary survey completed, FAST, X-ray c-spine, L leg, R elbow, CT, EKG	Found to have a L mid-shaft femur fracture and a R supracondylar fracture. No intra-abdominal fluid on FAST. No chest or intra-abdominal injury on CT. Laceration that requires suturing to R cheek. Abrasions to R hip and arm, contusions to L hip and thigh. No other injury. EKG unremarkable	Splints (L femur and R arm); antibiotics and tetanus. Take measures to keep temperature physiologic
Results of ordered tests	hemoglobin 116 hematocrit 0.45 platelets 250 white blood cell count 7.0 glucose 4.0 Na 135 Cl 90 K 4.0 CO2 24 AST 35 ALT 40 ALP 70 total bilirubin 16 amylase 70 INR 1.0 PTT 40 sec blood type A+	Get new set of vitals
Objective 3: Prepare for transport to a trauma center		
Stage 4: Prepare for transport		
Warm blankets, dressing to R cheek	Vitals stable	Call surgeon and arrange transport to trauma centre. Consider having a unit of packed red blood cells available during transport
If not adequate fluids	HR 140 // BP 75/50	Stabilize child then arrange transport to trauma centre. Consider having a unit of packed red blood cells available during transport
If not adequately resuscitated	HR 150 // BP 60/35 Child becomes drowsy	Stabilize child then arrange transport to trauma centre. Consider having a unit of packed red blood cells available during transport

Figure 1: Radiograph of left femur demonstrating a mid-shaft fracture (Source: K. Chan)

Figure 2: Radiographs of the right elbow demonstrating a supracondylar fracture (Source: K. Chan)

To ensure a smooth experience for trainees, an instructor completed a run-through of the scenario while acting as a trainee, prior to formal learning sessions. During a learning session, two instructors were present, one who maintained overall control of the scenario, and the second who took notes for the subsequent debriefing. 

Pre-Briefing

A pre-briefing was held with the trainees before the case. Limitations of the simulation were reviewed, in particular addressing technical issues with the mannequin and resource availability. The fiction contract agreement between participants and instructors to proceed as if the simulation is real while, simultaneously acknowledging it was not addressed [6]. Finally, trainees were advised that the case is strictly formative.

Case

In this simulation case, an eight-year-old male is brought to a community hospital after being struck by a pick-up truck as he ran across the road. His father, who accompanied the boy in the ambulance, saw the accident and said he did not think the boy lost consciousness. Upon request, trainees are provided with details of the patient's allergies, medications, and a past medical history that includes mild asthma. Learners were advised if an investigation was normal and/or were provided with a normal result from the simulation laboratory database.

At the beginning of the scenario, the patient is already connected to cardiac monitors with a full set of vital signs provided by emergency medical services (EMS) indicating an axillary temperature of 36.5 °C, tachycardia, and a blood pressure of 100/65. The scenario takes place in the trauma room of a community hospital with a full complement of resuscitation and intubation equipment on hand, as well as point of care ultrasound (PoCUS). Also available are crystalloid or colloid fluids, blood (O^-^ and type-specific), a rapid infuser, warming devices, splints (including Thomas), lab, x-ray, and computed tomography (CT). There are two registered nurses as well as a respiratory therapist working, and there is a general surgeon on call at the nearest trauma center.

The trainees are then instructed to proceed with their evaluation of the patient.

Debriefing

Following the conclusion of the scenario, trainees were provided with a formal debriefing. Care was taken during the debriefing to ensure that the number of debriefers is limited such that the debriefer-to-learner ratio does not exceed one: one. The debriefing is ideally lead by an educator with experience in debriefing; the principles of good judgment and frame discovery should be central to the process [7].

Post-Scenario Didactics

A didactic session was held after the debriefing. During this session, instructors were able to address any learning needs identified through the scenario and debriefs, and trainees were given the opportunity to strengthen knowledge gained through the simulation exercise. The following is meant to be a guide only, not a complete discussion.

Objective 1: Develop an approach to pediatric trauma: A general approach to trauma as outlined by the advanced trauma life support (ATLS) guidelines should be reviewed [8]. This provides the learner with a systematic, routine approach that prioritizes life and then limb threatening injuries. A thorough secondary survey can be conducted once the primary survey is complete and any life and limb threatening injuries are stabilized appropriately. Special consideration should be given to the mechanism of injury and the possible resulting injuries. In this case, suspicion for a femur fracture is high as this is common for children involved in an multi-voxel pattern analysis (MVPA). Chest and abdominal trauma must also be ruled out in any child hit by a vehicle [9]. It is also important for learners to recognize that children are likely to be reassured by the presence of a parent or caregiver and that this will diffuse some fear, enabling easier assessment and management of injuries. Of course, a parent or substitute decision maker is required to be involved for informed consent when emergent care will not be delayed to obtain consent. 

Objective 2: Recognize common pediatric injuries and their appropriate management: Although the basic tenets of trauma are the same for adults and pediatrics, special consideration should be given to a few differences. For example, c-spine injury patterns vary in young children due to anatomical differences; chest wall damage may be minimal despite significant pulmonary or cardiac injury due to rib cage compliance; abdominal solid organ injury is more likely in children in the setting of blunt trauma [9]. The educator should be familiar with pediatric trauma and therefore be able to highlight any knowledge gaps amongst the learners. 

Objective 3: Prepare for transport to a trauma center: The logistics of transportation from a regional center to a tertiary trauma center will vary from center to center. However, certain principles apply to all situations. Distance to the trauma center and the time required to mobilize transportation should be considered. Is ground or air transport available? Which is more appropriate to the geography, as well as to the urgency of the patient’s condition? It is important to ensure that staff trained to manage the severity of the patient’s injuries are available to accompany the patient. It is also important to ensure measures are taken to reduce morbidity or mortality during transport. For example, a patient may be electively intubated prior to transport if deterioration of the airway is anticipated during transport. The open femur fracture should be cleaned and dressed with minimal handling and then splinted before transport. The elbow fracture should also be splinted before transport. Also, fluid resuscitation, blood products, and analgesia should be available during transport. Ground transport via ambulance is a reasonable choice in this case, though a case could be made for air transport if the service cab can get the patient to the trauma center in less than 90 minutes. A physician escort is ideal, however, if no other physician in the community is available, royal navy (RN) escort would also be acceptable. A parent should accompany the child.

These issues should be discussed with the learner by the physician leading the didactic session. It is expected the learners are able to recognize when transport is necessary, and what preparation is required.

## Discussion

Teaching emergency medicine trainees how to manage pediatric trauma and prepare for transport to a designated pediatric trauma center through the use of a case simulation may be a valuable teaching tool.

In this simulation, key learning objectives for trainees include:

1. Develop an approach to pediatric trauma,

2. Recognize common pediatric injuries and their appropriate management,

3. Considerations for transport to a trauma center.

During the post-scenario didactic session, we considered the mechanism of injury and the importance of a thorough secondary survey. The logistics of transport was discussed. 

The development of the case scenario using a stepwise algorithm allows the simulation to react according to decisions made by trainees. Having an instructor complete the run-through both ensures that the case is of reasonable difficulty for the resident and enables instructors to address any limitations of the scenario. Finally, the use of a formal debriefing model coupled with a post-scenario didactic session allows instructors to identify and address not only knowledge gaps but also errors of process committed by trainees.

## Conclusions

Pediatric trauma can be very challenging and emotionally charged. Therefore, training in a controlled environment can be beneficial for postgraduate trainees and ultimately lead to better patient outcomes. We have presented a pediatric motor vehicle-pedestrian simulation scenario. An integrated teaching session incorporating simulation and didactics with components of debriefing targeted toward postgraduate medical learners is also presented.
